# Investigations of Metallurgical Differences in AISI 347 and Their Influence on Deformation and Transformation Behaviour and Resulting Fatigue Life

**DOI:** 10.3390/ma17184543

**Published:** 2024-09-16

**Authors:** Georg Veile, Elen Regitz, Marek Smaga, Stefan Weihe, Tillmann Beck

**Affiliations:** 1 Materials Testing Institute, University of Stuttgart, Pfaffenwaldring 32, 70569 Stuttgart, Germany; 2 Insitute of Materials Science and Engineering, RPTU Kaiserslautern-Landau, Gottlieb-Daimler-Straße 47, 67663 Kaiserslautern, Germany

**Keywords:** austenitic stainless steel, deformation induced martensite, delta ferrite, fatigue, metastability

## Abstract

Due to variations in chemical composition and production processes, homonymous austenitic stainless steels can differ significantly regarding their initial microstructure, metastability, and thus, their fatigue behavior. Microstructural investigations and fatigue tests have been performed in order to evaluate this aspect. Three different batches and production forms of nominally one type of steel AISI 347 were investigated under monotonic tensile tests and cyclic loading under total strain and stress control in low and high cycle fatigue regimes, respectively. The deformation induced α’-martensite formation was investigated globally by means of in situ magnetic measurements and locally using optical light microscopy of color etching of micrographs. The investigation showed that the chemical composition and the different production processes influence the material behavior. In fatigue tests, a higher metastability and thus a higher level of deformation induced α’-martensite pronounced cyclic hardening, resulting in significantly greater endurable stresses in total strain-controlled tests and an increase in fatigue life in stress-controlled tests. For applications of non-destructive-testing, detailed knowledge of a component’s metastability is required. In less metastable batches and for lower stress levels, α’-martensite primarily formed at the plasticization zone of a crack. Furthermore, the formation and nucleation points of α’-martensite were highly dependent on grain size and the presence of δ-ferrite. This study provides valuable insights into the different material behavior of three different batches with the same designation, i.e., AISI 347, due to different manufacturing processes and differences in the chemical composition, metastability, and microstructure.

## 1. Introduction

Austenitic stainless steels (ASSs) with high contents of chromium are popular due to their chemical resistance. With a chromium content of over 16 wt%, and austenite stabilizing elements like nickel, carbon, or manganese, they exhibit metastable austenitic microstructures at room temperature. AISI 347, also designated X6CrNiNb18-10, is an austenitic metastable Cr–Ni steel. In ASSs, chromium carbides tend to precipitate at the grain boundaries (GB). This reduces the Cr content in the austenitic matrix, and hence, reduces the chemical resistance close to the GB, which may promote intercrystalline corrosion [1]. Hence, niobium is added to AISI 347 as a carbide forming element with a high binding energy to carbon, thus avoiding chromium carbide formation at the grain boundaries and the associated Cr depletion. For this reason, AISI 347 is known for its resistance against intercrystalline corrosion and is used in applications in nuclear reactors in Germany if it matches the requirements of the German Nuclear Safety Standards Commission (KTA) [2]. As an opposite effect, the addition of certain elements like silicone reduces the austenite stability [3,4].

Plastic deformation or reductions in temperature can lead to a phase transformation in paramagnetic metastable fcc austenite into a more stable microstructure like hcp ε-martensite or ferromagnetic bcc α’-martensite [5,6]. Material properties like tensile or fatigue strength are influenced by this phase transformation. By measuring the magnetic properties, the formation of α’-martensite can be detected and thus its influence on the corresponding material properties can be observed [1,7,8]. With regard to non-destructive testing (NDT), measuring the content of deformation induced martensite (DIM) provides an opportunity. One of the earliest references regarding the α’-martensite detection via its magnetic properties was Scheil [9] in 1932. The DIM of various ASSs has been intensively studied in [1,7,10,11,12] with regard to metallurgical changes, fatigue behavior, or microstructural changes during deformation. In Ref. [12], the metastability of two batches of AISI 347 was investigated and compared. Variations in the chemical composition of two different batches notably influenced the metastability [12]. The chemical composition is known to influence the formation of DIM [13].

The authors of [8] investigated microstructural changes due to DIM under cyclic loading. They discovered that fatigue cracks in the microstructure often propagate at the phase boundaries between the original austenite and the newly formed α’-martensite. Crack propagation occurs mainly in the martensitic sub-grain structure with various different grain orientations [8,14]. In Ref. [14], researchers discovered the formation of α’-martensite before crack initiation and the formation of a martensitic-tunnel around a propagating crack. In contrast, the hypothesis of martensite as a preferential crack initiation site could was not supported in [15]. In Ref. [16], the metastable properties were investigated regarding their potential use for NDT methods due to the formation of α’-martensite and its impact on magnetic field variation in fatigue and strain increase tests.

**Figure 1:**
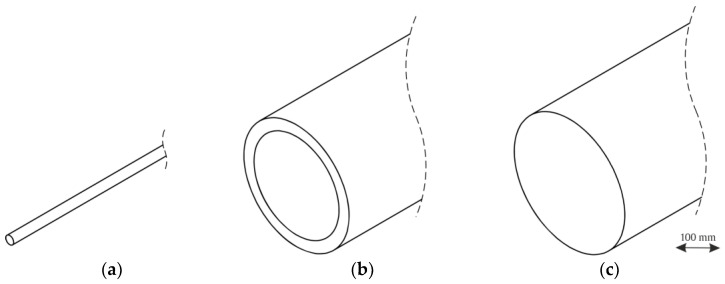
Investigated batches of AISI 347 stainless steel. (**a**) Standard rod scale 1:10; (**b**) Pipe (surge pipe line) scale 1:10; and (**c**) Shaft with modified scale 1:10. Batches (**b**,**c**) were previously owned by nuclear power plants and match KTA standards.

In this work, three different batches of AISI 347 were analyzed: a standard rod, pipe, and shaft (Figure 1). Each batch matched the standard of DIN EN 10088 [17,18] but had different chemical compositions. All investigated batches were formed by different production processes, such as being hot rolled, wound with a piercing mill, or forged. Two of these batches (Pipe and Shaft) were in accordance with the requirements of KTA. While KTA 3205.1 [19] specifies requirements for components with non-integral connections to the primary coolant circuit and orientates with [17,18] regarding the chemical composition, KTA 3201.1 [20] specifies components in contact with reactor water over 200 °C. AISI 347 materials matching the specifications of KTA 3201.1 are given the material number EN 1.4553 and are designated as X6CrNiNb18-10S, coming with comparably lower carbon contents (Table 1). The letter “S” at the end of the material designation does not stand for a chemical element. The origin is not described in the datasheet. X6CrNiNb18-10S is also defined in the material data sheet of VdTÜV 451 [21] for components in the primary circuit of light water reactors and the external components of pressurized water reactors. The latter defines the δ-ferrite content in the weld penetration area of the forged X6CrNiNb18-10S base metal with regard to the application of welding without filler metal between 2–10% and 1–10% with filler metal. A closed δ-ferrite network is ineligible [21]. The available KTA-certified materials are unique; they are no longer in production due to Germany’s decision to stop nuclear powered energy production by the end of 2022 [22], but are still in use in South Korean nuclear power plants. 

The chemical composition, microstructure, heat treatments, and production processes of various batches impact the occurrence of DIM during mechanical loading. Due to this sensitivity and the impact of α’-martensite on the material behavior of metastable austenitic stainless steels, it is of interest to analyze the occurrence of α’-martensite. The transformation of α’-martensite due to plastic deformation is induced at a temperature rage between M_s_ ≤ T ≤ M_d_. Hence, ASSs are described with values such as the martensite start temperature M_s_, M_d30_, or stacking fault energy (SFE). The SFE is given in mJ/m^2^ [4,23,24,25,26]. M_d30_ is the temperature in °C where 30% plastic strain results in 50 vol% of α’-martensite [3,4,13,27,28]. M_s_ is the martensite start temperature in °C for thermally induced α’-martensite formation [3,4,27]. The relevant characteristic values regarding the metastability can be calculated with the chemical composition using Equations (1)–(3), where the letters represent the content of the elements.

(1) $$M_{S,Eichelmann}=1350-1665\left(C+N\right)-28Si-33Mn-42Cr-61Ni$$

(2) $$M_{d30,Angel}=413-462\left(C+N\right)-9.2Si-8.1Mn-13.7Cr-9.5Ni-18.5Mo$$

(3) SFE=2.2+1.9Ni−2.9Si+0.77Mo+0.5Mn+40C−0.016Cr−3.6N

Studies on the metastability of ASS were primarily carried out by the research group led by Smaga et al. [1,12,29], also regarding nuclear grade AISI 347 [1,12]. The focus of these works was the analysis of various methods for NDT, e.g., in [12], the influence of chemical inhomogeneities was studied, while [11] focused on metallurgical differences among various ASS with special regard to metastability. The aim of this work is to analyze metallurgical differences in AISI 347 and their influence on the deformation and transformation behavior, as well as the resulting fatigue life, due to different production processes and standards. To achieve the defined goal, the chemical composition of the batches and its impact on metastability were investigated. Furthermore, quasi-static tensile tests, fatigue tests (total strain- and stress-controlled), and impact tests were conducted. The microstructure was investigated using EBSD and color etching of micrographs.

## 2. Materials and Methods

### 2.1. Materials

The investigated batches of AISI 347 were delivered in three different shapes (Figure 1): firstly, a generally available rod (hereafter called Rod) with a diameter of 25 mm matching the requirements of DIN EN 10088 [17,18], delivered by Lämmermeier Edelstahl GmbH; secondly, a surge line pipe (hereafter called Pipe) with a diameter of 333 (± 2) mm and a wall thickness of 36 mm, produced by DMV stainless Deutschland GmbH and in accordance with the high requirements of the KTA; and finally, a forged shaft (hereafter called Shaft) with a diameter of 350 (±1) mm of X6CrNiNb18-10S, produced by BGH Edelstahl Siegen GmbH, also meeting the high requirements of KTA 3201.1. 

The geometry and production processes of the initially delivered materials and their specific heat treatments are listed in Table 2. The chemical compositions are given in Table 3. The addition of alloying elements in KTA certified materials, such as nickel and niobium, were identified by spectral analysis. ASSs are known for their two-stage phase transformation from fcc austenite to hcp ε-martensite and then bcc α’-martensite [6,28,30,31,32]. Since a transformation into hcp ε-martensite is not be expected with a low carbon content, it can be assumed that all DIM is α’-martensite [8]. The mechanical properties and initial conditions, such as hardness measurement, conducted according to DIN EN ISO 6507, were determined in this work and are listed in Table 4 with standard deviations. The FeritScope^TM^ has a measurement accuracy of ±0.01 FE-%.

### 2.2. Determination of Susceptibility to DIM

Characteristic values like SFE, M_s_, or M_d30_ do not consider the manufacturing history of the material. For example, the stress–strain state in the specimen caused by cold hardening plays an important role regarding the formation of α’-martensite but is not considered in SFE, M_s_, or M_d30_. In Ref. [12], a method that takes into account the chemical composition and the history of the material was introduced. These tests were conducted with metastable austenitic steels of type AISI 304, 321, 348, and also two batches of 347. There, magnetic measurements were conducted at room temperature (RT) on a plastic deformed area of a flat specimen. Local plastic deformation was induced with a dynamic hardness test (Poldi hardness tester with 10 mm Brinell ball). The affected area had sufficient size to equalize local deviations in the chemical composition. The magnetic measurements after local mechanical loading (I_ξ_) conducted with a Feritscope^TM^ provided an impression of the specimen’s susceptibility to DIM.

### 2.3. Tensile Tests

**Figure 2:**
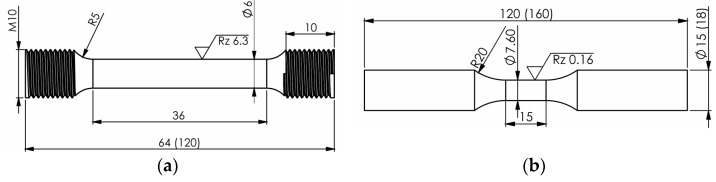
Specimen geometry for tensile (**a**) and fatigue (**b**) tests.

The specimen geometry of tensile tests is visualized in Figure 2a. To measure the occurrence of DIM in situ in relation to strain and stress, a Feritscope^TM^ was applied on the specimen, and the stress, strain, and ferromagnetic fraction ξ were recorded. For this reason, the ferromagnetic fraction is visualized in FE-%. All measured α’-martensite fractions will be indicated in FE-% due to the limitation of the linear correlation between ferrite and α’-martensite under 60 vol% [3,34]. The tensile tests were carried out on an electromechanical testing machine of type Z250 SN, manufactured by ZWICK GmbH Co.KG, with a rated force of ±250 kN according to the standard of DIN EN ISO 6892-1. A strain rate of 2.5·10^−4^%s^−1^ was applied until yield strength, followed by 6.7·10^−3^%s^−1^ until failure. The test was conducted at RT.

### 2.4. Fatigue Tests

The cyclic deformation and transformation behavior were investigated under total strain-control in the low cycle fatigue (LCF) regime and under stress control in the high cycle fatigue (HCF) regime. Therefore, a servo-hydraulic testing system was used to apply a triangular waveform. A Feritscope^TM^ measured the ferromagnetic fraction ξ in situ. The geometry of each fatigue specimen is illustrated in Figure 2b and was identical for all batches regarding the measuring area in terms of gauge length. The surface of the fatigue specimens was mechanically and electrolytically polished in order to remove DIM due to the manufacturing processes. This was conducted in Struers Elektrolyte A3 I (methanol, 2-butoxyethanol, perchloric acid 60%).

Strain-controlled fatigue tests were conducted with a total strain amplitude ε_a,t_ of 1% and total strain ratio of R_ε_ = −1 at a frequency of 0.01 Hz. The comparably low frequency led to a temperature increase below 1 K. Stress-controlled fatigue tests were conducted with an amplitude of σ_a_ = 280 MPa at a frequency of 2 Hz and a stress ratio R_σ_ of −1. The tests were based on the experimental design of [12] to realize a practical duration of testing. The fatigue tests were conducted at RT. The metastability of the austenitic microstructures is highly dependent on the temperature, with a decrease of DIM at increased temperatures [12]. Due to the testing frequency, a maximum specimen temperature of 65 °C only for the short test time (up to 1000 loaded cycles/500 s) was measured, which did not influence the formation of DIM to a significant degree.

### 2.5. Microstructure Investigations

After common metallurgic preparation of the micrographs, material was removed from the surface using electrolytic polishing to avoid DIM due to mechanical polishing. If necessary, the polishing process was conducted at an increased temperature (90 °C) to avoid the occurrence in DIM in the initial state due to abrasion. Furthermore, etching by the Beraha-II agent was used to distinguish the individual grains, their orientation, the austenite and α’-martensite, as well as chemical inhomogeneities [10] in the micrographs. For more details of the process, see [35]. Additionally, electron backscatter diffraction (EBSD) images of the grain orientation were generated using a scanning electron microscope (SEM) of the type QUANTA 600 FEG, manufactured by FEI. There, an accelerating voltage of 20 kV at a step size of 1 with a view field of 300 µm was applied. For analyses of the specimen microstructure after failure, investigations were undertaken using Beraha-II etching in the initial state and after the tests. The resulting color of the etched micrographs varied due to variations in the preparation process.

## 3. Results

### 3.1. Microstructure in the Initial State

**Figure 3:**
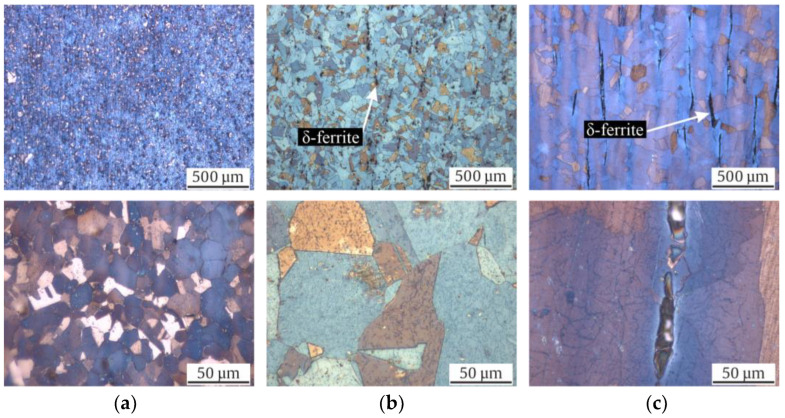
Light optical micrographs of the initial microstructures of (**a**) Rod (**b**) Pipe and (**c**) Shaft in longitudinal sections.

**Figure 4:**
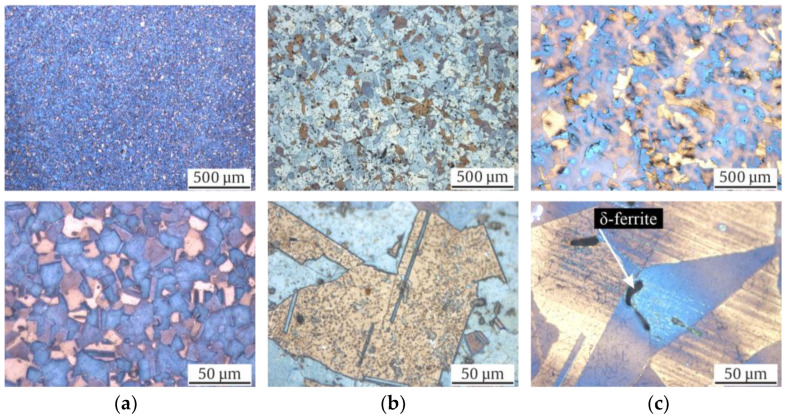
Light optical micrographs of initial microstructure of (**a**) Rod (**b**) Pipe and (**c**) Shaft in cross section.

**Figure 5:**
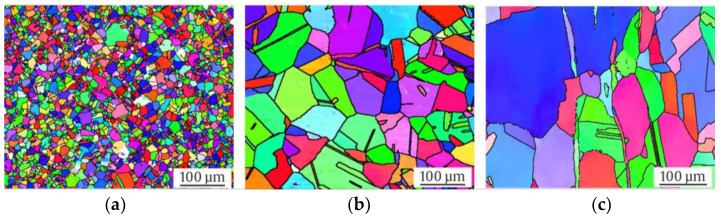
EBSD micrographs of the initial microstructures of (**a**) Rod (**b**) Pipe and (**c**) Shaft in cross section.

The microstructures of the investigated materials in their initial states are shown as longitudinal micrographs in Figure 3 and as cross sections in Figure 4. All three micrographs and the EBSD images of Figure 5 show a typical austenitic microstructure with twins.

Using Beraha-II color etching, one can observe inhomogeneities in the chemical composition in the Rod (Figure 3a and Figure 4a) and Shaft (Figure 3c and Figure 4c). Elongated grains due to the forging production process are seen in Figure 3c. However, the Pipe, which was widened in a piercing mill, did not show comparable chemical inhomogeneities.

Note that the mean grain size of Pipe and Shaft were comparable with 120 (±14) and 153 (±56) µm, while the Rod material showed a significantly smaller grain size of 17 (±9) µm (Table 4). The largest grain could be measured in the Shaft with 521 µm, followed by 240 µm in the Pipe, and lastly, 40 µm in the Rod. Furthermore, no α’-martensite could be observed visually with its needle shaped formation in all investigated batches. A striking difference was the shape of the black δ-ferrite. Whilst micrographs of the Shaft material showed the typical sausage-shaped formation of δ-ferrite, the Pipe material showed δ-ferrite, which was more discontinuous in the longitudinal direction and expanded in the cross-sectional direction. Since the micrographs showed no α’-martensite in the initial state, it could be concluded that the ferromagnetic fraction (Table 4) measured using the Feritscope^TM^ was δ-ferrite. Moreover, the sausage-shaped δ-ferrite occupied more volume in the illustrations of the Shaft material compared to the Pipe material. This confirmed the previously outlined observation, since only in the Shaft material was a ferromagnetic fraction of 2.9 FE-% measured, while the Rod showed 0.0 FE-% and the Pipe 0.0 to 0.3 FE-%. Furthermore, the distribution of the δ-ferrite in the microstructure of the Shaft material was not homogeneous. The Rod material showed no δ-ferrite in its initial state. This was further confirmed by the Feritscope^TM^ measurements (Table 4).

### 3.2. Suscepibility to DIM

**Figure 6:**
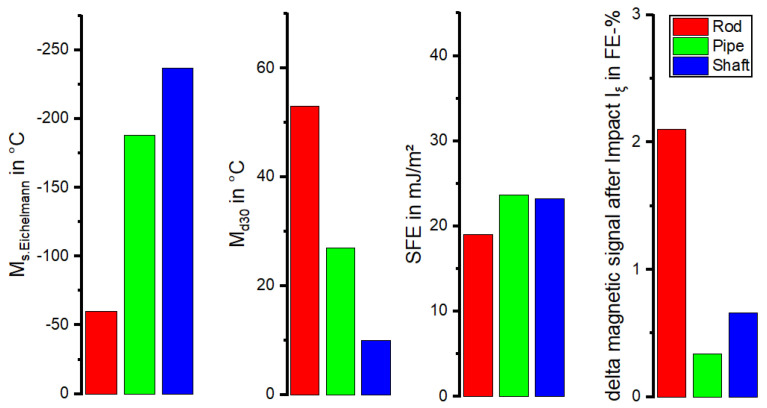
Characteristic values describing the metastability of the investigated materials, such as M_s_, M_d30_, or SFE, and the difference of measured FE-% after the impact loading.

The metastability of austenitic stainless steels is known to be highly correlated with the chemical composition. Our metastability assessment based on chemical composition (Table 3) was conducted using the empirically derived Equations (1)–(3) of M_s_ and M_d30_ temperatures, as well as the SFE. The results are illustrated in Figure 6. 

All materials show a SFE of over 20 mJ/m^2^. The characteristic values M_s_ and M_d30_ indicated that the Rod material was the most metastable of the three batches. With the M_S_ temperature of −60 °C, the Rod batch transformed at higher temperatures compared to the Pipe (−188 °C) and Shaft (−237 °C) batches. Furthermore, the M_d30_ of the Shaft batch with 10 °C and Pipe with 27 °C was lower than the 53 °C of the Rod material. 

Among the three materials, Rod exhibited the highest metastability owing to a distinct minimum in M_s_ temperature and SFE and a maximum in M_d30_ temperature. This conclusion is supported by the impact loading and subsequent magnetic measurements of the ferromagnetic α’-martensite fraction (I_ξ_), which showed a significant higher value for the Rod material compared to the other two materials.

On the other hand, no conclusive statements could be made regarding the metastability of the more stable materials, i.e., Pipe and Shaft. While the M_s_ and M_d30_ temperatures suggested that Pipe was more susceptible to DIM than Shaft, both batches exhibited a similar SFE level. In contrast, the I_ξ_ value suggested a higher metastability for the Shaft material. However, it should be noted that the magnetic field generated by the Feritscope^TM^ coil may have interfered with the magnetic field of the pre-existing ferromagnetic δ-ferrite in the Shaft material and the ferromagnetic DIM, which may have led to interference in the measurements. Additionally, the variation of the δ-ferrite content of the Shaft material in the initial state was significant due to the diffuse distribution of the δ-ferrite throughout the material volume, resulting in inconclusive observations after impact loading. Hence, it can be assumed that for materials containing δ-ferrite in the initial state, the aforementioned methods may not provide a clear indication of susceptibility to DIM.

### 3.3. Monotonic Loading and Deformation Induced α’-Martensite Formation

**Figure 7:**
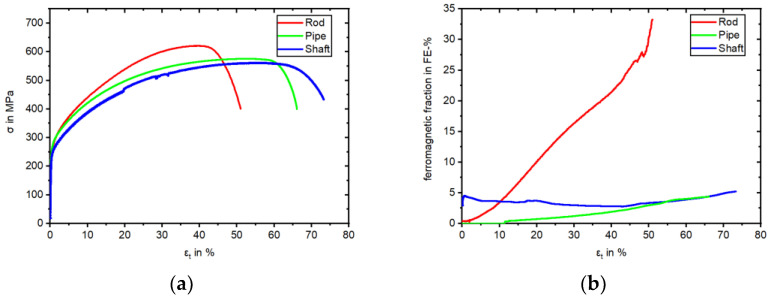
Stress–strain (**a**) and magnetic fraction–strain response (**b**) from tensile tests.

The results of the tensile tests are presented in Table 4 for comparison of the three batches. The resulting stress–strain curve is illustrated in Figure 7a with the corresponding evolution of the measured ferromagnetic fraction in Figure 7b. 

Starting from zero, the Rod specimen formed up to 33.2 FE-%, due to the formation of DIM. The applied strain to the now partly bcc lattice of the specimen led to a tensile stress of 621 MPa at 39% strain. Fracture of the Rod specimen occurred at 51%. One could observe the continuous rise of DIM with applied strain. The same applied to the Pipe specimen, albeit to a lesser extent, with 4.4% FE-% This resulted in a comparably lower tensile stress of the mostly fcc lattice. Both KTA certified materials (Shaft and Pipe) showed a similar maximum stress, stress–strain curve, and higher fracture elongation. However, the measured magnetic signal of the Shaft specimen deviated from the other two. Within the first 2% strain, the signal rose to 4.9 FE-%, starting from 2.9 FE-% at 0% strain. With continuous applied strain, the signal declined to 2.8 FE-% at 37% strain, followed by rising to 5.2 FE-% at fracture. The major difference was the δ-ferrite content in the initial state of Rod and Pipe compared to Shaft (Table 4) and the correlation with this progression of the magnetic signal.

### 3.4. Cyclic Deformation Behavior and Deformation Induced α’-Martensite Formation in LCF and HCF Regime

**Figure 8:**
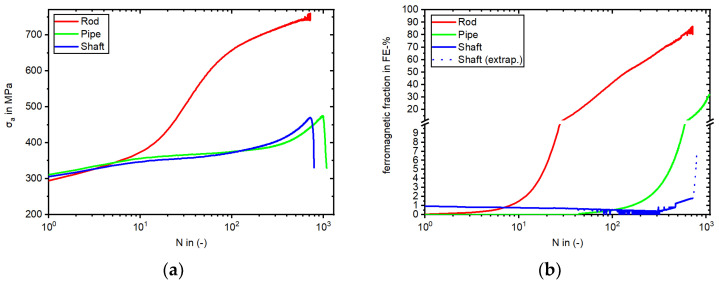
Development of stress amplitude- (**a**) and deformation-induced α’-martensite formation (**b**) versus the number of cycles during LCF tests; ε_a,t_ = 1.0%, R = −1, f = 0.01 Hz.

The cyclic deformation behavior and the development of α’-martensite during LCF tests with a total strain amplitude of 1% is shown in Figure 8. The Rod specimen started from a stress amplitude under 300 MPa, whilst the Shaft and Pipe started above 300 MPa in the first cycle. Regarding the Rod specimen, the increase of stress was inherent with the increase of measured α’-martensite starting after five cycles. The stress amplitude of the Rod specimen showed no descent and failed rapidly at 718 cycles, which could be attributed to the position of the extensometer relative to the crack. The Rod’s stress rose to 746 MPa, surpassing the UTS of the tensile test, corresponding with the increase of the measured ferromagnetic fraction due to α’-martensite with 85 FE-% at failure. In this specimen, the formation of DIM was detected after five load cycles. The stress amplitude of the specimens taken from the Pipe and Shaft differed compared to the Rod batch. KTA certified materials showed a nearly identical stress amplitude development but differed in terms of the measured ferromagnetic fraction. While the Pipe started to develop measurable α’-martensite after 30 cycles, the specimen of the Shaft showed a similar development as described in the tensile test. At this point, is must be noted that the applied in situ measurement with the Feritscope^TM^ to the Shaft specimen failed with crack initiation and the resulting decrease in stress. Hence, this specific ferromagnetic fraction is visualized as a dashed line starting from 700 cycles, approaching the manual measured fraction after failure with 6.7 FE-%. The Pipe specimen endured 1084 cycles with 31.6 FE-%, while the Shaft specimen failed at 791 cycles.

**Figure 9:**
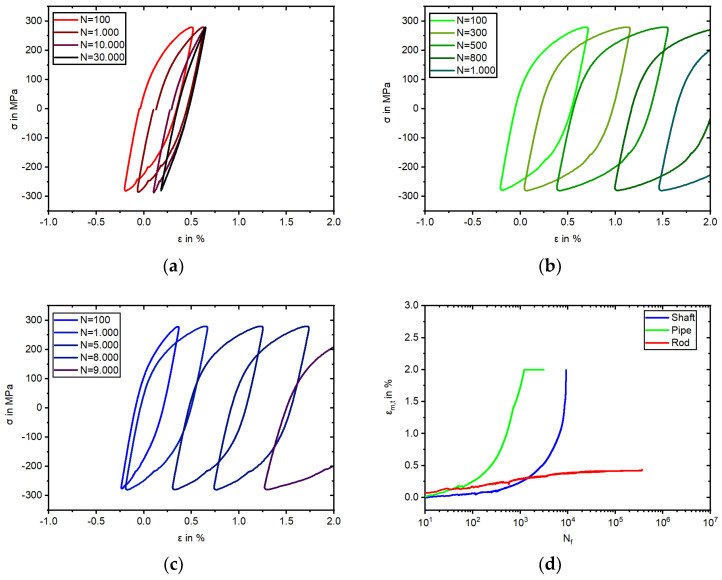
Stress–strain hysteresis loops of Rod (**a**) Pipe (**b**) and Shaft (**c**) and (**d**) total mean strain in HCF tests with σ_a_ = 280 MPa, R = −1, f = 2 Hz.

**Figure 10:**
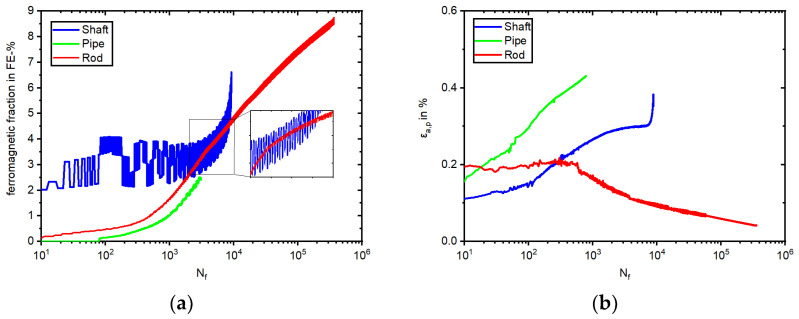
Development of (**a**) α’-martensite and (**b**) the plastic strain amplitude during HCF tests with σ_a_ = 280 MPa, R = −1, f = 2 Hz.

The results of the stress-controlled fatigue tests are illustrated in Figure 9 and Figure 10. The experiments were conducted with a stress amplitude of 280 MPa at a stress ratio of R_σ_ = −1. In contrast to the total strain-controlled experiments, an increased frequency of 2 Hz was chosen for the stress-controlled experiments to obtain a high number of load cycles within a reasonable time and temperature increase. All three batches showed cyclic ratcheting in the stress-controlled fatigue test, and thus, an increase in the total mean strain (ε_m,t_) (Figure 9d), and with this, the movement of the hysteresis loops to the higher positive strains (Figure 9). For Shaft and Pipe, this behavior was more pronounced than for Rod, ultimately exceeding the measuring range of the extensometer for the Pipe at 1209 cycles and Shaft at 9150 cycles (cf. Figure 9b,c). For the Rod, the cyclic ratcheting reached a saturation at around 10500 cycles within the measuring range of the extensometer, and the ε_m,t_ did not increase significantly from this point. 

The Pipe specimen failed first at 3065 cycles, followed by the Shaft specimen at 9196 cycles. Finally, the Rod specimen failed at 365,018 cycles. Besides the cyclic ratcheting behavior (Figure 9), the development of DIM and plastic strain amplitude (ε_a,p_) differed for the three batches (Figure 10).

The change in the magnetic Feritscope^TM^ signal is illustrated in Figure 10a. Starting at 0.0 FE-% and ending with 8.7 FE-%, the Rod specimen showed the highest increase, starting rapidly at 1000 cycles. The specimen taken from the Shaft started with 2.0 FE-% due to the presence of δ-ferrite and showed, besides measurement changes in the Feritscope^TM^ signal due to the Villari effect, an increase to 6.6 FE-% at failure. In contrast, the Pipe material showed a similar signal development to the Rod specimen with no initial measured ferromagnetic fraction and failed at 2.5 FE-%.

In Figure 10b, one can observe the initial softening behavior for all three batches. The Pipe softened more than the Shaft, as the Shaft contained significantly more δ-ferrite. For the Pipe material, softening continued until the measuring range of the extensometer had been exceeded. The Shaft specimen tended to transition to saturation at 3200 cycles, correlating with the measured increase in the ferromagnetic fraction in Figure 10a. For the Rod, cyclic softening was not so pronounced, whereby the minimal not closed hysteresis loops (Figure 9a) influenced the development of the plastic strain amplitude, followed by hardening with increasing DIM at 570 cycles.

### 3.5. Microstructure after Failure

**Figure 11:**
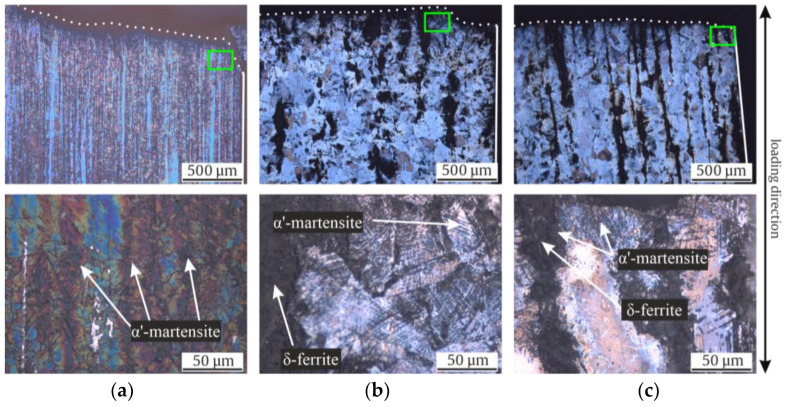
State of the microstructures at the breaking edges after failure due to total strain-controlled fatigue tests of Rod (**a**), Pipe (**b**), and Shaft (**c**). Crack initiation starting from the right side and propagating to the left.

Analyzing the material microstructures after failure gave us the opportunity to evaluate the local formation of α’-martensite due to the applied loads. With the same process of color etching, the polished sections showed not only different fractions of α’-martensite, but also different nucleation points of DIM. Figure 11 depicts the etched micrographs after failure in the total strain-controlled fatigue tests with cross sectional polished sections. The area of fracture initiation was chosen to be investigated more in detail with the micrographs. 

**Figure 12:**
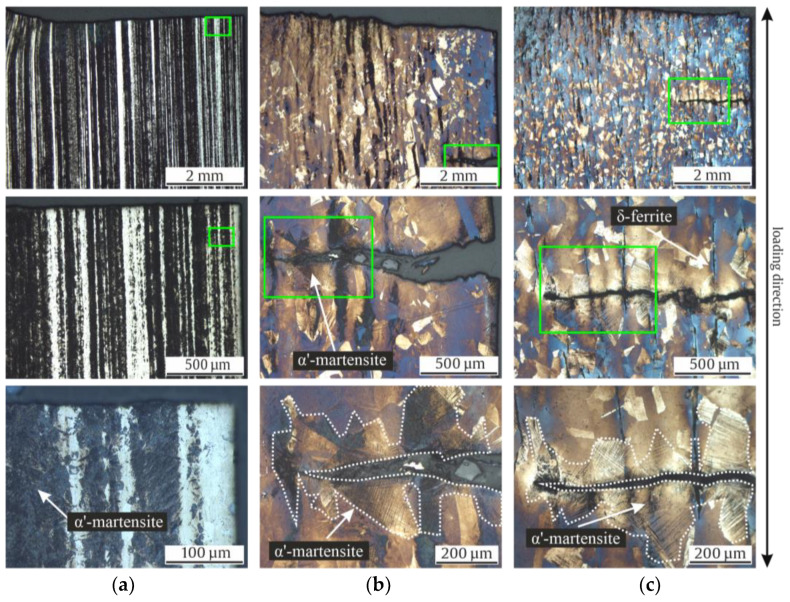
State of the microstructures after failure of Rod (**a**), Pipe (**b**) and Shaft (**c**) due to stress-controlled fatigue tests.

Figure 12 shows the resulting micrographs after stress-controlled fatigue tests. The Pipe and Shaft specimens show a minimal localized increase of α’-martensite around the cracks. However, in the KTA certified materials (Pipe and Shaft), most of the α’-martensite formed around the present δ-ferrite. In Figure 12b,c, it can be seen that DIM did not form homogeneously with continuous crack growth due to chemical inhomogeneities, visualized with the color etching. While all specimens show α’-martensite in the load direction, the Rod material showed a more significant quantity, although no δ-ferrite was present in the material in its initial state.

## 4. Discussion

The micrographs in the initial state show how much homonymous materials can differ in terms of their microstructure, grain size (Table 4), production process, and thus, metastability. Notably, only the KTA-certified materials (Pipe and Shaft), named X6CrNiNb18-10S, contained δ-ferrite; the δ-ferrite content was significantly higher for Shaft. The presence of δ-ferrite is due to the requirements, as described in [21], for nuclear grade materials. The formation of a δ-ferrite phase in austenitic stainless steels has been investigated in numerous studies, which have mostly noted that it is formed during solidification, while a few stated that it forms during hot deformation [36]. The increased amount of δ-ferrite could be attributed to the higher chromium content in the batch of the Shaft and, to a smaller extent, the comparably lower Cr content compared to the Pipe [5]. Variation of δ-ferrite quantity in the microstructure could furthermore be associated with the cooling rate decreasing toward the core [37]. The shape of the δ-ferrite is affected significantly by the production process. While the δ-ferrite in Shaft showed a typical sausage-like morphology, for the Pipe material, it displayed a deformed structure due to the widening process in a piercing mill. With respect to the grain size (Table 4), Pipe and Shaft resembled each other’s microstructures, with 120 and 153 µm equivalent grain diameters, respectively, due to the identical heat treatment temperature and the similar chemical compositions (Table 3). In contrast, micrographs of the Rod material with a smaller grain size of 17 µm showed no δ-ferrite, resembling the material investigated in [16]. The color etching of the micrographs with Beraha-II revealed chemical inhomogeneities in the form of chemical banding in Rod and Shaft, visible in the longitudinal section (Figure 3a,c), due to the manufacturing process [10]. As described in [10], these inhomogeneities have already been documented in Cr-Ni type ASS and have an impact on DIM due to local variations in chemical composition. This parameter also has an impact on values like M_s_, M_d30_, and SFE (see Equations (1)–(3)). In contrast to Rod and Shaft, the chemical components in Pipe were distributed more evenly. It has to be mentioned that the metallographic sample preparation poses a particular challenge, especially for highly metastable ASS, as a low level of phase transformation has to be ensured [38]. Acosta et al. [16] and Donnerbauer et al. [39] found a microstructure with α’-martensite inside a X6CrNiNb18-10 Rod material in their experiments and attributed this to the forming process at low temperatures with heat input applied to the rod’s surface. In our work, the same phenomena was observed in micrographs in samples of highly metastable stainless steel (SFE about 20 mJ/m^2^) ground at RT; however, grinding at elevated temperatures, i.e., above 90 °C, yielded the presented, free of preparation-induced martensite micrographs. So, in this case, abrasion was the causative of the observed DIM.

It is worth mentioning that the authors of [40,41] published investigations of AISI 304L, depicting minimal fractions of ε-martensite after loading. However, in other previous works [1,12,27,42], no hcp ε-martensite was detected in AISI 347 alloys like those investigated in this work. This could also be confirmed with the EBSD of the Shaft material after the stress-controlled fatigue test. Differences in metastability could already be identified in the initial state of the materials based on the chemical composition. Accordingly, these differences also influenced the material behavior during monotonic and cyclic loading. Looking at the tensile and fatigue tests conducted in this work, as well as the micrographs, it can be said that the Rod was the most metastable. This resulted in a higher UTS of 621 MPa but a comparably smaller elongation at fracture (Table 4) compared to the Pipe and Shaft materials. Both the Pipe and Shaft showed a similar progression of stress and elongation at fracture, which highlights the consistency of the two KTA certified materials. Regarding the metastability, the latter was followed by the Pipe, while the Shaft material showed the least tendency to DIM. This correlated with the individual increase of the magnetic signal in the total strain-controlled (Figure 8b) and stress-controlled fatigue test (Figure 10a). This finding was supported by the of M_d30_ and M_S_ values (Figure 6) which, hence, yielded a good prediction regarding metastability. These results are basically consistent with the findings in [1,3,7,12,27,41,43], where these values were compared for various metastable austenitic stainless steels. But as seen in the example of SFE, the values were, in this case, not consistent, due to the different weighting of the chemical elements in the empirical equations, i.e., for M_d30_ and M_S_, elements C and N were weighted equally, while this is not the case for SFE. In contrast, a prediction of the metastability was only successful with the calculated SFE and I_ξ_ measurement for the Rod material, while the relation of Pipe and Shaft metastability was not predicted correctly, as the Shaft material was less metastable in the tensile and fatigue tests. It is noteworthy that the I_ξ_ values were very close to each other regarding the Pipe and Shaft material, and therefore, it is not yet appropriate to speak of a clear tendency, even though these values were the medians of five individual measurements. In Refs. [1,12], SFE and impact loading gave a good prediction of the metastability of AISI 347. However, the microstructure described in [1] did not show δ-ferrite as in the micrographs of the Pipe and Shaft materials in this work. Furthermore, the measurements presented in [1,12] started at 0.0 FE-%. The measurement with the Feritscope^TM^ for the impact test may have been influenced by the magnetic properties of δ-ferrite. In Ref. [12], no δ-ferrite was present. For this reason, it is likely that the presence of δ-ferrite in the microstructure influenced the validity of the SFE and I_ξ_ value. The micrographs after the fatigue tests, i.e., Figure 11b,c as well as Figure 12b,c, show that δ-ferrite served as a sprouting point for DIM, which increased the effect.

The sensitivity of the measurement with the Feritscope^TM^ to δ-ferrite can also be seen in the measured ferromagnetic content of the tensile test (see Figure 7b) of the Shaft. As described in the results, after a short increase, the measured amount decreased. The Rod and Shaft material did not show comparable behavior, having no or less δ-ferrite, in accordance with [29]. Combined with the above analysis, it is very likely that the δ-ferrite impacted the measurement of DIM with the Feritscope^TM^. References [44,45,46] mention different magnetic properties of δ-ferrite and α’-martensite.

Considering the cyclic deformation behavior in the total strain-controlled tests, the development of the stress amplitude of the Rod material differed significantly compared to the batches with KTA certification. While all three batches showed an increase due to cyclic hardening and failed at a comparable number of cycles, the specimen taken from the Rod material surpassed even the initial ultimate strength of 621 MPa, as determined in tensile tests (Figure 8a and Table 4). Similar to our observation of the Rod specimen, fatigue tests presented in [3,7,41] also surpassed the initial ultimate strength due to the formation of α’-martensite. It is worth mentioning that in [7], this only occurred for materials with the highest metastability and not in all alloys, and is thus in accordance with the findings of this work comparing the resulting stress of all three batches. After rapidly reaching 600 MPa, the rising magnitude of the stress amplitude gradient of the Rod specimen declined to an almost constant value until failure (Figure 8a). This can be attributed to the depicted deformation-induced α’-martensite formation (Figure 8b) and the concomitant change from a fcc to a bcc lattice providing a higher stress at the same strain level. The in situ measured ferromagnetic fraction surpassed 60 FE-% at 223 cycles. At this point, it must be noted that over 60 FE-%, the Feritscope^TM^ lacked measurement linearity between δ-ferrite and α’-martensite. Further explanation and detail are given in [1]. Nevertheless, the depicted sigmodal shape (Figure 8b) was in accordance with the results of [3] but did not develop to completion, since not every material reached saturation before failure, thereby showing only the formation of α’-martensite, which cannot generally be declared as evidence of failure for metastable ASS using NDT. Furthermore, the same α’-martensite content (e.g., 2.15 FE-%) could be measured at different fatigue states (1452, 2444, and 275 cycles), dependent on the metastability of the homonymous materials which, can be seen in Figure 10a. As already mentioned in the results of this work, it was striking that although both KTA materials showed a similar progression of the stress amplitude (Figure 8a), with a cyclic hardening due to DIM and δ-ferrite and a resulting failure at comparable numbers of cycles, the measured Feritscope^TM^ signal illustrated in Figure 8b differed. However, by considering the microstructure of the both specimens after failure, a similar quantity of α’-martensite in Figure 11b,c could be observed, showing that the magnetic measurement did not display the development of DIM in the Shaft material correctly. As discussed in the context of the determination of the I_ξ_ values, the measurement with the Feritscope^TM^ should be viewed with caution if δ-ferrite is present in the initial state of the material. 

The tendency toward metastability of the three batches continued in the stress-controlled fatigue test. In the micrographs, after the test in Figure 12, it can be seen that the Rod material displayed the most α’-martensite. The pipe material formed significantly less α’-martensite compared to the Rod material, as described in the results of this work. The δ-ferrite-rich Shaft material formed even less (Figure 12c), as discussed in the previous section. If one compares the stress evolution during the total strain-controlled fatigue test, which reached >450 MPa before failure with a 280 MPa stress amplitude in the stress-controlled fatigue test, it is evident that the lower load led to less DIM. As mentioned in the results, the Shaft specimen showed more changes compared to the Rod and Pipe specimens in the in situ magnetic measurements with the Feritscope^TM^ (Figure 10a). This phenomenon can also be seen in [47], coming with an explanation based on the change of magnetic susceptibility caused by mechanical stress, known as the Villari-effect. This influences the ability to determine when DIM occurs during an experiment. It is noteworthy that the development of the ferromagnetic signal for Shaft (Figure 10a) correlated with the magnetic signal of the Shaft in the tensile test (Figure 7b), showing an increase in FE-% in the beginning, followed by a decrease and a secondary increase until specimen failure due to the presence of δ-ferrite in the microstructure. This phenomenon was also observed by Hauser et al. [48] but was not described or mentioned. In summary, it can be noted that the formation of α’-martensite in Figure 10a in the stress-controlled fatigue test was in accordance with previous findings of the materials’ metastability, where Rod was the most metastable material batch, followed by the Pipe and then the Shaft material.

Considering the cyclic deformation behavior, within the first approx. 300 cycles, all three batches showed a cyclic softening behavior, whereby the cyclic softening for Rod was not as pronounced as for Shaft and Pipe. The difference in magnitude of the initial softening of all specimens may have been related to the different grain sizes, where the Rod material with 17 µm grain size showed the least softening. Shaft also showed less softening compared to Pipe; this may have been due to the higher content of bcc δ-ferrite in the initial state (Table 3). After approx. 300 cycles, reaching 0.7 FE-%, Rod showed a significantly different material behavior than Pipe and Shaft, as it underwent cyclic hardening, and the specimen was able to undergo the fatigue tests with a stress amplitude of 280 MPa with a lower plastic strain amplitude (see Figure 10b) compared to the other two batches. The authors of [6,49] cited plastic strain-induced martensitic phase transformation as a possible cause of secondary hardening. The origin of this material behavior was studied in [50,51,52,53], also at elevated temperatures, and is not the focus of this work. The pronounced cyclic hardening in this work cannot be explained only by the high metastability of Rod alone, since an increase in DIM of 0.7 FE-% did not lead to a cyclic hardening for Pipe and Shaft, even though Pipe showed a similar development of DIM as Rod. At the beginning of the test, a decrease in the FE-% signal was observed in the Shaft before the α’-martensite increase started, which was also shown in reference [48]. Furthermore, due to the Villari-Effect a different change in the FE-% signal occurred at the same mean value in dependency of the present δ-ferrite and α’-martensite content. Both had to be taken into consideration for this measurement. One possible explanation for the different material behaviors of the three batches may be that the interaction between α’-martensite and material dislocations for materials with smaller grain sizes (e.g., Rod with 17 µm) was more pronounced than in materials with larger grain sizes (e.g., 120 µm of Pipe and 153 µm of Shaft). This is also a possible explanation for the variation in the ratcheting, as shown in Figure 9a–c. This theory is in accordance with the literature [54,55]. However, in Ref. [56], fatigue tests conducted with asymmetrical stress cycling yielded the opposite result. Although Shaft had the biggest grain size, and one could assume that it would also show the highest cyclic softening, the presence of δ-ferrite influenced the material behavior, leading to a less pronounced cyclic softening than for the Pipe specimen. Comparing the hysteresis loops area of the Pipe specimen (Figure 9b) with the area of the Shaft specimen (Figure 9c), one can clearly see that the specimen taken from the Pipe material exhibited more strain energy density and thus suffered more damage to the specimen integrity. The sequence of plastic strain amplitude (see Figure 10b) corresponded to the failure sequence of the samples, where the Pipe specimen failed first, having the highest plastic strain amplitude, followed by the Shaft specimen. 

Comparing the numbers of cycles at failure with the results of stress-controlled fatigue tests in the literature, one can observe the striking difference in life expectancy for AISI 347. The Pipe specimen failed at 8732 cycles, i.e., close to the 7000 cycles of the AISI 347 tested by [12]. However, the Shaft material exceeded this with 18957 cycles, even though the stress amplitude was 5 MPa higher compared with the experiments published in [12] (also undertaken at 2 Hz). In Ref. [29], AISI 347 was tested at 5 Hz for stress amplitudes of 280 to 225 MPa, also taken from a Ø 25 mm Rod material. Furthermore, in Ref. [29] only a specimen with a stress amplitude of 250 and 225 MPa reached 2×10^6^ cycles, forming 2 and 6 FE-% DIM. The specimen taken from the Rod material in this work, with its high metastability, reached 365,018 cycles at 280 MPa stress amplitude, resulting in 6.6 FE-% However, the specimen in [29] under 280 and 270 MPa did not reach 10^5^ cycles, having >7.5 FE-%. This comparably earlier failure at an identical stress amplitude of 280 MPa may have been due to the frequency of 5 Hz compared to the 2 Hz used in this work. The influence of frequency on the fatigue of AISI 347 was investigated in [12], where a specimen with 0.2 Hz endured more cycles compared to specimen with 2 Hz applied frequency. 

With a view to the microstructure, in Refs. [8,42], the development of “martensitic tunnels” is described due to cyclic loading, and the resulting plastic zones that formed at the crack tips, where the local stress had a higher magnitude compared to the nominal stress. In Refs. [8,42,57], the crack tip of a non-failed specimen were analyzed. This facilitated the analysis of the crack flank for the preparation of the micrographs compared to our work, where broken specimens were analyzed. According to our observations, these striking martensitic tunnels were not seen in the micrographs after failure in total strain-controlled fatigue loading (Figure 11). However, the dark colored α’-martensite increased toward the crack flank in Figure 11b,c, with increased DIM around δ-ferrite. In addition, this was also confirmed with micrographs of the stress-controlled fatigue tests specimen in Figure 12b,c. Nevertheless, analyzing a martensitic tunnel of cracks in a broken specimen is less expedient as the methods in [8,42,57], i.e., analyzing cracks that have not separated the specimen. In this work, an increased quantity of α’-martensite can be observed at the secondary cracks in Figure 12b,c. The amount of dark colored α’-martensite increased at the edges of the crack, propagating toward the center of the specimens, confirming the findings in [8,35,42,58]. According to the observations of Sohrabi et al. [40], analyzing AISI 304, the nucleation sites of α’-martensite were shifted with increasing grain size from the grain boundaries to the interior of the grains. DIM primarily formed at the grain boundaries of the Rod micrographs (Figure 11a and Figure 12a), with its smaller grain size of 17 µm, and inside the grains of the Pipe micrographs, with a grain size of 120 µm (Figure 11b and Figure 12b). This is in accordance to the findings in [40] regarding the nucleation based on the grain size of ASSs.

It is noteworthy that α’-martensite primarily started to form at the δ-ferrite of the Shaft material (see Figure 11c) in the total strain-controlled fatigue test. Since the Pipe micrographs after failure did not show α’-martensite exclusively, but also inside the grains (Figure 11b), δ-ferrite could not be identified as causative for this striking local formation of DIM. However, one could assume that the shape of δ-ferrite influences the local sensitivity to DIM. If this assumption is correct, one could expect more α’-martensite at the crushed δ-ferrite parts of the Pipe material (Figure 3b), since the local stresses in the microstructure of the material should be higher there than in the rounded, sausage-shape δ-ferrite of the Shaft material (Figure 3c). Since this assumption was not confirmed, the shape of δ-ferrite may not have been responsible for the local occurrence in the Shaft material. Another possible explanation is the local difference in chemical composition, which can also be explained by the manufacturing processes (Table 2) and the fact that δ-ferrite absorbs chromium, sulfur, and phosphorous [49,59]. Chemical inhomogeneities can be visualized with the color etching process. Man et al. [10,11] found local chemical variations in the form of chemical banding. This can be seen to a significant extent in the micrographs of the Shaft material in Figure 3c, with a seemingly luminous blue tone around the δ-ferrite compared to the Pipe material in Figure 3b, which did not show chemical banding after the color etching process. For a well-founded causal analysis, however, an identical melt of the austenitic material would be necessary as a basis for a comparison, since the volume fraction of the δ-ferrite of the two samples differed, as seen in the measured initial fraction in Table 4. This identical melt would have to be used for the different production processes of Pipe and Shaft (as described in Table 2) to achieve a different form of the δ-ferrite, as one can observe in the micrographs of the initial state in Figure 3b,c. Nevertheless, according to the work of Neidel et al. [60], subsequent processing can decrease the volume fraction of δ-ferrite. Considering the production process of Pipe, this is an additional possible cause for the comparably lower initial ferromagnetic fraction, although Pipe and Shaft had undergone a similar heat treatments (Table 2) and had similar chemical compositions (Table 3). Nevertheless, with regard to the chemical banding in the initial state (Figure 3a,c), i.e., etched with Beraha-II, and the formed α’-martensite in areas after the fatigue tests (Figure 11a,c and Figure 12a,c), the results are consistent with [11].

The discussion of the results shows that the non-destructive testing of metastable materials with in situ magnetic measurement should be treated with caution, especially when δ-ferrite is present. The chemical composition and the calculation of the SFE did not consider the production process of the materials. Furthermore, the measured values of the Feritscope^TM^ were influenced by the presence of δ-ferrite, and therefore, have to be taken into account to make a statement on the material’s metastability based on I_ξ_ measurements. Color etching is a good tool to show the metastability and local inhomogeneities due to the production process. The micrographs after the fatigue tests were excellent for analyzing and interpreting the results from other measurement methods. The three investigated batches effectively show how sensitive metastable austenitic materials are due to their chemical composition and production processes. The results generated in this work are largely consistent with those in other publications and provide a deeper understanding of AISI 347, specifically X6CrNiNb18-10 and X6CrNiNb18-10S. However, they also show how different the material behavior of homonymous alloys can be under identical test conditions.

## 5. Conclusions

The study illustrates the sensitivity of the mechanical behavior of AISI 347 ASS to metallurgical variability, phase composition, and production techniques. Our results align well with the existing literature but underscore the complexities in predicting material behavior due to differences in metastability and production-related factors. It can be stated that metastable ASS, specifically, the so called AISI 347, also known as X6CrNiNb18-10 and X6CrNiNb18-10S, differs regarding its material behavior under monotonic and cyclic loading due to DIM. Considering the results, the subsequent conclusions can be made:

- Micrographs reveal differences in microstructure, grain size, and metastability due to the production process. Notably, KTA-certified materials show variations in δ-ferrite content and morphology, influenced by the production process and chemical composition.
- Chemical inhomogeneities in all batches impacted DIM due to local composition variations, especially close to δ-ferrite. Microstructural post-fatigue tests highlighted the role of δ-ferrite in α’-martensite formation and its impact on DIM, as well as material behavior under cyclic loads. With comparable fatigue life, microstructural responses varied due to the presence of δ-ferrite.
- All investigated ASSs showed a SFE conducive to a direct transformation from γ-austenite to α’-martensite.
- Material exhibiting high metastability shows higher ultimate tensile strength but lower elongation at break. In contrast, material with lower metastability showed consistent stress and elongation patterns.
- In all strain-controlled fatigue tests, materials with high metastability surpassed initial strength estimates. Softening and hardening material behavior was shown to be influenced by DIM and grain size.
- The presence of δ-ferrite challenges the accuracy of NDT like Feritscope^TM^, influencing the measurement of magnetic properties during mechanical testing. For the NDT, with a Feritscope^TM^, exact knowledge of the material’s metastability and component operation conditions have to be known. Matching an identical material standard (e.g., DIN or KTA) is not sufficient, since the metastability may vary. If one aims to make a statement about the integrity of a component by investigating the presence of α’-martensite, one has to be aware of the initial state of the material, its tendency to DIM, and its load history.

## Figures and Tables

**Table 1:** Requirements for chemical composition of the investigated metastable austenitic stainless steel.

Standard	C	Si	Mn	Mo	P	S	Cr	Nb	Ni	Co	N	Ti
KTA 3201.1	≤0.04	≤1	≤2	-	≤0.035	≤0.015	17 ≤ & ≤ 19	10 × C ≤ & ≤ 0.65	9 ≤ & ≤ 12	≤0.2	-	-
KTA 3205.1/EN10088	≤0.08	≤1	≤2	-	≤0.045	≤0.015	17 ≤ & ≤ 19	10 × C ≤ & ≤ 1.00	9 ≤ & ≤ 12	-	-	-

**Table 2:** Overview of delivered products with the corresponding production processes and specific heat treatments.

Batch or Geometry	Production Process	Heat Treatment	Standard
Rod	hot-rolled and peeled to a diameter of Ø 25 mm	Solution annealed at 1050 °C for 35 min in vacuum, quenched in helium	DIN
Pipe	hot-rolled, widened in a piercing mill, turned on the outside (Ø 333 mm), and drilled out inside; wall thickness of 32 mm	Manufactured according to to DIN 17458 [33]. Solution annealing temperature: 1050 °C; holding time for 10 min; water quenched	KTA
Shaft	forged and turned to a diameter of Ø 350 mm	Solution annealed, 1050 °C holding time for 1 h, water quenched	KTA

**Table 3:** Chemical composition in wt% of all AISI 347 batches determined by spectral analysis.

	C	Si	Mn	Mo	P	S	Cr	Cu	Ni	Nb	N
Rod	0.025	0.56	1.57	0.16	0.03	0.006	17.0	0.19	8.91	0.41	0.027
Pipe	0.040	0.41	1.83	0.29	0.02	0.007	17.6	0.06	10.6	0.62	0.007
Shaft	0.031	0.38	1.94	0.40	0.03	0.003	18.1	0.14	10.6	0.54	0.034

**Table 4:** Mechanical properties and initial condition state parameters of the investigated AISI 347 batches.

	E-Modulus	R_p0.2_	UTS	ε_fracture_	Grain Size	HV10	Initial ξ
Rod	202 (±12) GPa	220 (±1) MPa	621 (±3) MPa	51 (±0.3) %	17 µm	152 (±6)	0.00 FE-%
Pipe	196 (±0.6) GPa	231 (±0.7) MPa	576 (±13) MPa	66 (±5.3) %	120 µm	140 (±5)	0.0-0.3 FE-%
Shaft	196 (±0.8) GPa	227 (±0) MPa	562 (±1) MPa	73 (±0.7) %	153 µm	142 (±3)	1.8-2.9 FE-%

## Data Availability

Raw or processed data maybe shared upon request to the corresponding author after approval of the MPA and RPTU directors, the project executing agency (GRS) and German Federal Ministry for Environment, Nature Conservation, Nuclear Safety and Consumer Protection.
